# Systemic Vasculitis Post-COVID-19: A Case Report

**DOI:** 10.7759/cureus.72724

**Published:** 2024-10-30

**Authors:** Shamsun Nahar, Mohammad M Husain, Akshay Maharaj, Mohit Lakkimsetti, Yashwanth Vemoori, Mohammad Nazmul Karim, Shaila S Saaki, Farhana Binta Awal Oeshe, Rokeya Begum

**Affiliations:** 1 Internal Medicine, California Institute of Behavioral Neurosciences and Psychology, Fairfield, USA; 2 Internal Medicine, Kansas City University of Medicine and Biosciences, Fort Myers, USA; 3 Internal Medicine, Port of Spain General Hospital, Chaguanas, TTO; 4 Internal Medicine, Mamata Medical College, Khammam, IND; 5 Internal Medicine, Rajarajeshwari Medical College and Hospital, Bengaluru, IND; 6 Internal Medicine, Bangladesh College of Physicians and Surgeons, Dhaka, BGD; 7 Internal Medicine, Dhaka Medical College and Hospital, Georgetown, USA; 8 Internal Medicine, Bangabandhu Sheikh Mujib Medical College, Faridpur, BGD; 9 Internal Medicine, Jalalabad Ragib-Rabeya Medical College, Sylhet, BGD

**Keywords:** antineutrophil cytoplasmic antibody (anca)-associated vasculitis (aav), complications, covid-19, pathophysiology, treatment choices, vasculitis, vasulitis

## Abstract

Vasculitis is one of the complications of COVID. Coronavirus may trigger or exacerbate autoimmune diseases, such as systemic vasculitis. We present the case of an elderly individual with a medical history that includes recurrent urinary tract infections, hyperlipidemia, essential hypertension, and peripheral vascular disease. Doctors suspected vasculitis due to declining renal function, clinical condition, and serological findings of the patient. His serum tested positive for antinuclear antibodies. A kidney biopsy was deemed necessary due to the unclear cause of his renal insufficiency. The biopsy revealed focal necrotizing and crescentic glomerulonephritis (GN), a pauci-immune type. It is essential to learn more about COVID-19 and its related complications. This case highlights the difficulties of COVID-19, leading to focal necrotizing and crescentic GN. Maintaining a broad differential is essential while treating a patient who has recovered from the initial infection. This research is important because it will help clinicians to identify this perspective while treating patients. We also review some related articles on the association of COVID-19 with vasculitis.

## Introduction

COVID-19, also known as coronavirus or severe respiratory syndrome (SARS-CoV-19), is a multisystem inflammatory disease [1]. The severity of the infection varies, ranging from mild or moderate to severe illness, depending on the patient’s age and previous comorbidities. The lungs are the most affected organs, but other organs such as the kidneys can also be involved. Reports indicate that the incidence of acute kidney injury (AKI) in hospitalized COVID-19 patients ranges from 0.5% to 80% [2-4]. Antineutrophil cytoplasmic antibody (ANCA)-associated vasculitis is associated with granulomatous and neutrophilic tissue inflammation and the production of antibodies against neutrophil antigens. The condition may lead to renal failure through mononuclear cell infiltration, which destroys small- and medium-sized blood vessels. Severe acute respiratory syndrome (SARS) may exacerbate autoimmune diseases [5,6]. Glomerulonephritis (GN), although rare, can occur as a result of moderate to severe COVID-19 and may develop gradually or suddenly.

## Case presentation

An 83-year-old Caucasian man began experiencing poor feeding, malaise, fatigue, and generalized weakness for 10 days, with his spouse reporting that he would sway off balance. Four days after these symptoms began, he tested positive for COVID-19 using an at-home test. Although prescribed Paxlovid, he never started the medication. The patient also began to develop mild confusion in the following days. Ten days after the onset of his symptoms, he visited the emergency department, where a second COVID-19 test confirmed his positive status. Notably, the patient did not exhibit shortness of breath, chest pain, nausea, vomiting, diarrhea, or urinary symptoms.

His medical history includes an episode of urinary retention six months prior, complicated by benign prostatic hyperplasia (BPH) diagnosed 15 years previously, which was treated with holmium laser enucleation of the prostate (uro-surgical procedure). This admission marks his second admission related to COVID-19, with the first occurring 15 months earlier. The patient has not received a COVID-19 vaccine, and he uses continuous positive airway pressure to treat his obstructive sleep apnea. His medical history also includes cerebral infarction; heart failure with preserved ejection fraction; recurrent urinary tract infections, including methicillin-resistant *Staphylococcus aureus* (MRSA); shingles; hyperlipidemia; essential hypertension; right bundle branch block (RBBB); peripheral vascular disease; anemia; and cataracts, which were surgically treated. The patient reports drinking seven glasses of wine per week and has a history of 20 years of smoking, having quit 14 years ago. His family history includes a stroke on the paternal line and cancer in a sibling. He did not report any family history of renal disease. Upon observation, the patient appeared well, speaking and ambulating without issue. His vital signs recorded are as follows: blood pressure: 141/95 mm Hg, pulse: 86 beats per minute, respiratory rate (RR): 16 breaths per minute, temperature (T): 98.6 C, and SpO_2_: 98% on room air. Lung auscultation revealed rhonchi, and a chest X-ray showed left infra-hilar pneumonia. A head computed tomography (CT) was negative for acute processes such as stroke or hydrocephalus. He was dehydrated with mild hyponatremia (Na: 133), and his creatinine (Cr) was 1.86 on admission, compared to a previous baseline of 0.88. The blood urea nitrogen/creatinine (BUN/Cr) ratio was normal.

The urine culture grew MRSA; however, blood and sputum cultures were negative. The complete blood count showed neutrophilic leukocytosis (white blood cell: 22.3 x10³/µL), and the erythrocyte sedimentation rate was 77 in the first hour. The doctors started the treatment with Rocephin and Azithromycin. Despite comprehensive conservative management, including four days of intravenous fluids, the patient developed oliguria and his Cr levels rose to 5.06. He refused urinary catheterization. The renal ultrasound did not show any hydronephrosis or hydroureter but did show increased echogenicity of the bilateral kidneys, compatible with medical renal disease, and thickening of the urinary bladder wall. The doctors started treatment with Daptomycin and performed emergent hemodialysis four days into the admission, after which the Cr decreased to 2.63. In addition, a renal biopsy was performed due to the unknown etiology of renal failure and nephrotic range proteinuria. The biopsy revealed focal necrotizing and crescentic GN, pauci-immune type. The treatment plan included Rituximab, pulse IV solumedrol 500 mg daily for three doses, and avoidance of nephrotoxins.

The patient's AKI was likely multifactorial such as it can be related to poor oral intake and COVID-19 infection. However, the presence of nephrotic range proteinuria and hematuria coupled with the recent COVID-19 infection raised concerns for acute GN. The patient's vasculitis panel showed a positive proteinase 3 antibody and antineutrophil cytoplasmic antibody (C-ANCA) with a titer greater than 1:640. All myeloperoxidase, antinuclear, and glomerular basement membrane antibodies were negative. Complement levels were normal. Tests for hepatitis, human immunodeficiency virus (HIV) panel, rapid plasma reagin, antiphospholipid A2 receptor antibody, serum protein electrophoresis, serum immunofixation, and serum-free light chains were unremarkable.

**Figure 1:**
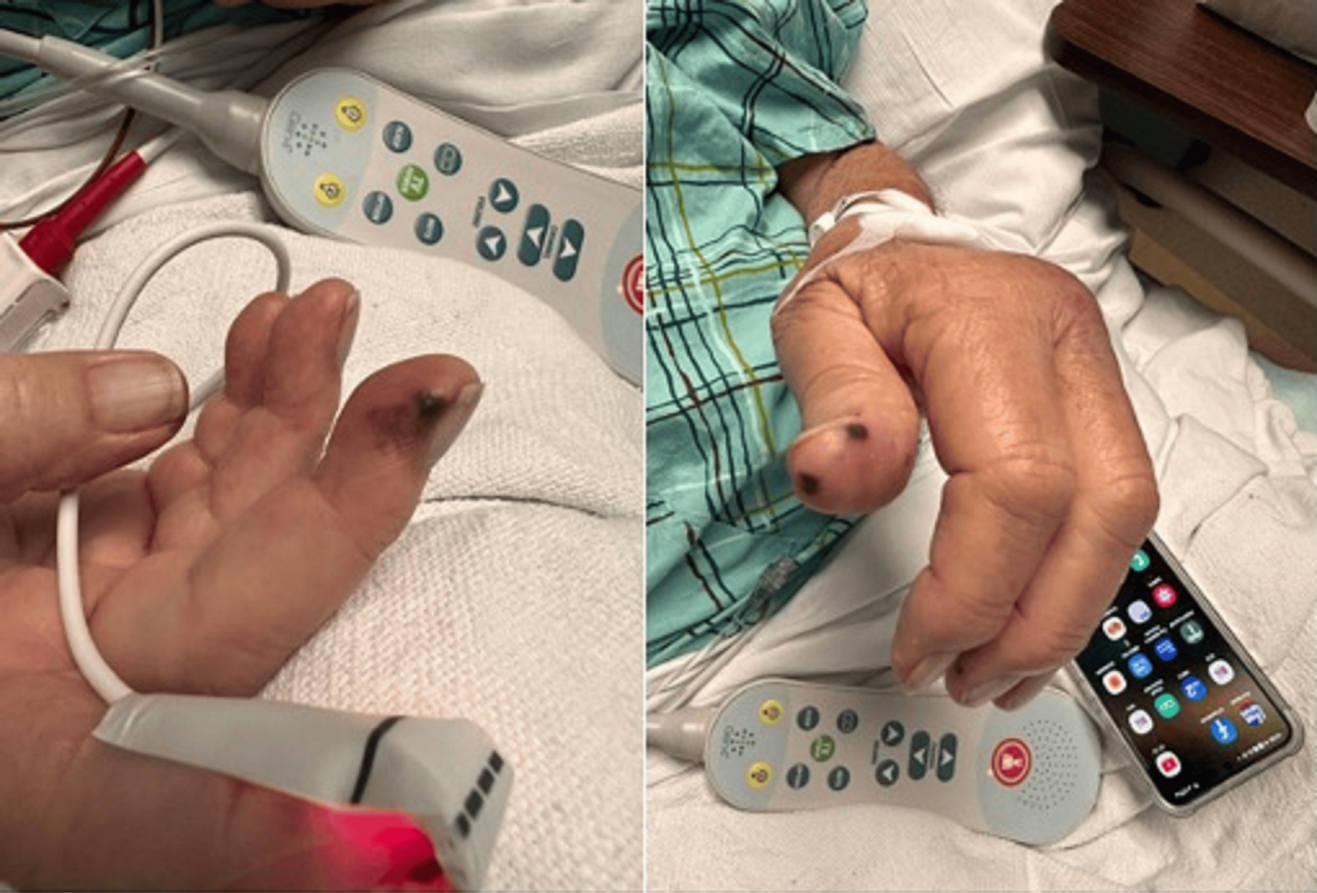
Hemorrhagic macules over both hands

One week into his hospitalization, the patient began to experience shortness of breath with minimal exertion. A chest X-ray showed bilateral infiltration with focal opacification of the left lung and a small pleural effusion. A CT scan of the chest showed bibasilar atelectasis. After a pulmonology consultation, the patient was diagnosed with acute hypoxic respiratory failure secondary to severe acute respiratory syndrome coronavirus 2 (SARS-CoV-2) infection, and the bilateral pleural effusion was likely due to volume overload. The patient was already receiving Decadron and Heparin; doctors added bronchodilators to improve airway clearance. After hemodialysis, his shortness of breath improved, and his oxygen requirements decreased. Additionally, the patient complained of painless hemorrhagic macules over both hands, as illustrated in Figure 1.

Doctors performed a transthoracic echocardiogram to rule out endocarditis, which showed no vegetation. Following a consultation with an infectious disease specialist, the team concluded that the vascular lesions on the hands were likely related to hypercoagulability associated with COVID-19. Due to elevated D-dimer levels, prophylactic anticoagulation was initiated. A ventilation-perfusion scan was done to rule out a pulmonary embolism and showed low probability. A Doppler kidney ultrasound showed no recent vein thrombosis or renal artery stenosis, and a venous duplex ultrasound showed no evidence of deep vein thrombosis.

The urine microscopy showed 11-20 WBC/high power field, RBC >50, 0-2 hyaline cast, with no bacteriuria or epithelial cells. The echocardiogram (ECHO) with contrast showed an average left ventricular (LV) end-diastolic dimension, LV ejection fraction of 57%, Grade 1 LV diastolic dysfunction, mild concentric LV hypertrophy, and no significant changes compared to prior ECHO. The electrocardiogram (EKG) showed sinus rhythm, normal P axis, ventricular rate between 60 and 99, RBBB, and QRS complex >120. The results of other investigations are summarized in Table 1.

**Table 1:** Results of investigations ordered

Investigation Name	Result	Reference Range
Prothrombin time (PT), partial thromboplastin time	14, 37	10-13 seconds
C reactive protein (CRP)	4.4	0.3-1.0 mg/dL
Albumin and albumin/globulin ratio (A/G)	1.0	1.1-2.5
Alkaline phosphatases	145	44-147 IU/L
Creatine kinase	<20	22-198 U/L
Brain natriuretic peptide	2,630	<100 pg/mL
Urine analysis - specific gravity	1.012, significant proteinuria 100, and hematuria	1.005-1.030
Protein/Cr	271	<0.2 in adults
Urine sodium (Na)	52 mmol/L	40-220 mEq/L
Fractional excretion of sodium (FeNa)	>1.08	<1.0%

Upon discharge, the patient was started on a weekly Rituximab schedule for a total of four doses, which was then tapered to six monthly doses. After completing IV solumedrol 500 mg, the patient was started on oral prednisone at 60 mg daily, gradually tapering every four weeks to 40 mg daily. He also received atovaquone for Pneumocystis jiroveci pneumonia prophylaxis.

One month after discharge, the patient presented with complaints of multiple loose stools for five days. He was admitted due to AKI resulting from dehydration, with his Cr increasing to 1.9 mg/dL from baseline. A CT scan of the abdomen and pelvis was performed, showing results compatible with colitis and postoperative changes in the bladder region.

He was initially treated with vancomycin. Afterward, Fidoxomycin was added, which eventually resolved his symptoms. Plans were made to administer a repeat dose of Rituximab and reduce the prednisone to 10 mg after one month. However, he was readmitted one month later with complaints of flank pain and diarrhea. A repeat CT scan of his abdomen and pelvis revealed sigmoid colitis. Infectious disease specialists recommended continuing oral vancomycin and scheduling a Bezlotoxumab infusion post-discharge. Additionally, he experienced generalized weakness and shortness of breath, with bilateral pulmonary infiltrates identified in investigations. Physical therapy was provided, and IV Lasix was administered accordingly. Furthermore, he was diagnosed with a rhinovirus infection, which improved with symptomatic treatment. The patient is currently clinically stable, and we summarize his most recent investigation results in Table 2.

**Table 2:** Most recent investigation results The table contains the patient's lab results, which are included in the article with permission from the patient/hospital.

Investigation Name	Results	Reference Range
Na	138	135-144 mmol/L
Potassium	4.7	3.5-5.1 mmol/L
Chloride	107	98-110 mmol/L
CO_2_	20	22-30 mmol/L
Anion gap	11	4.0-12 mmol/L
Glucose	78	70-100 mg/dl
BUN	22	6.0-24 mg/dl
Cr	1.17	0.58-1.30 mg/dl
BUN/Cr ratio	18.8	7.0-25.0
Calcium	9.8	8.5-10.5 mg/dL
Protein, total	6.1	3.5-9.0 g/dL
Phosphorus	3.7	2.8-4.5 mg/dL
Albumin	3.2	3.4-5.4 g/dL
Globulin, total	2.4	2.0-3.5 g/dL
A/G ratio	1.1	1.1-2.5
Alkaline phosphatase	111	38-126 U/L
Aspartate aminotransferase	28	8-33 U/L
Alanine aminotransferase	15	7-56 U/L
Bilirubin, total	0.4	0.1-1.2 mg/dL
Magnesium	3.0	1.7-2.2 mg/dL
Lipase	99	10-140 U/L
Glomerular filtration rate	>60	≥60 mL/min/1.73 m^2^

## Discussion

Definition

The general definitions of vasculitis and GN are the inflammation of blood vessels and the kidneys' glomeruli, respectively. Various factors, including inflammatory diseases, genetics, malignancies, drugs, and infections, such as COVID-19, can cause GN [7]. GN can manifest in different types, and vasculitis is an essential cause of GN, which can also belong to other classes. Vasculitis occurs when inflammatory white blood cells cause reactive harm to the internal structures of blood vessels [8]. In vasculitis, there is necrosis of the vessel wall due to tissue ischemia from lumen compromise and bleeding from vessel wall disintegration [8]. ANCA-associated vasculitis is a subcategory of vasculitis involving small blood vessels [8].

Pathophysiology

**Figure 2:**
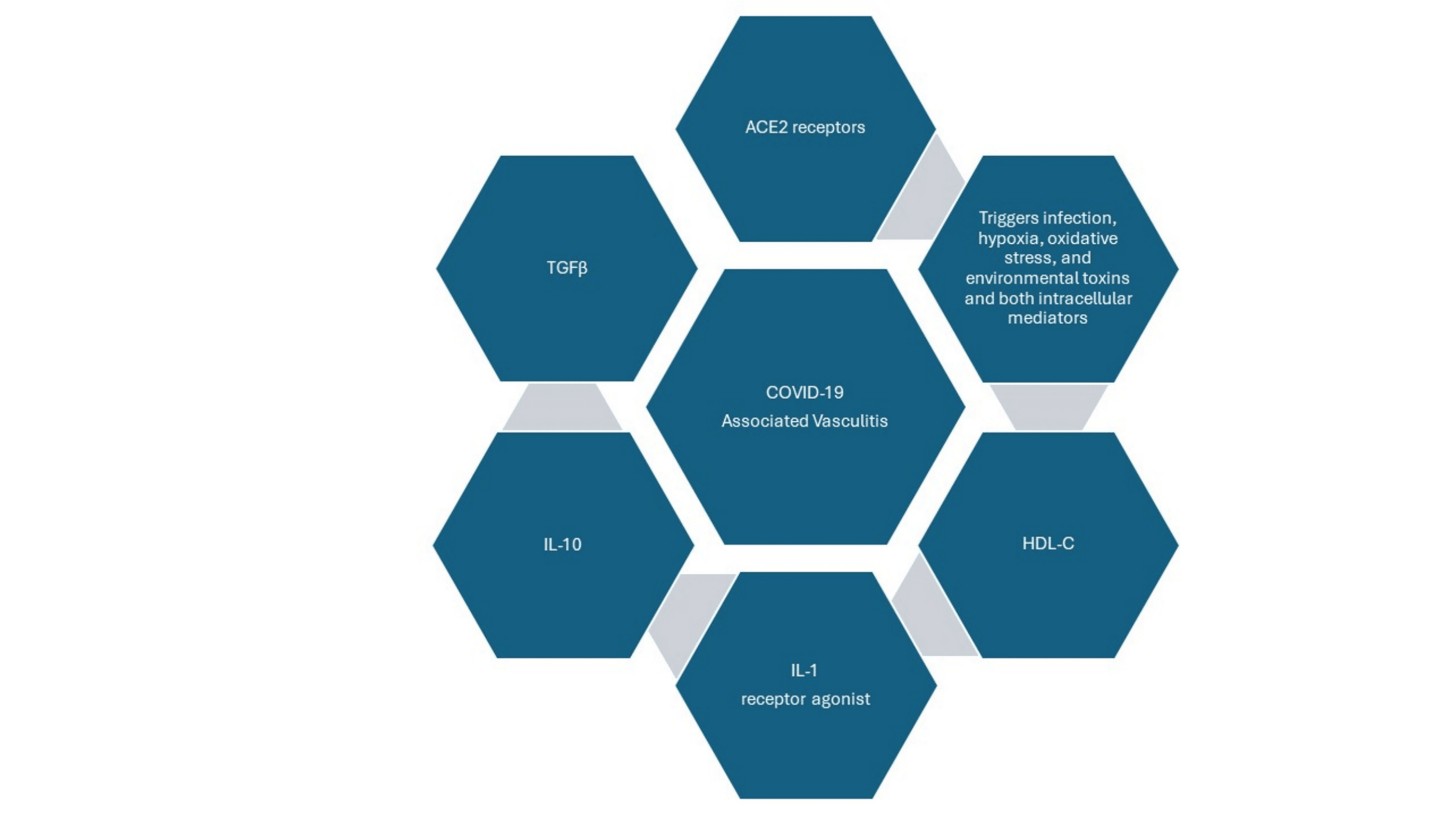
Triggers, signals, and mediators of COVID-19-associated vasculitis Image Credits: Shamsun Nahar

COVID-19 and ANCA-associated vasculitis share identical pathways, such as neutrophil extracellular traps, which induce complement activation and endothelial dysfunction [9]. The alternative complement pathway in ANCA-associated vasculitis and COVID-19 infection involving the C5aR1 receptor plays a crucial role [9]. It can cause endothelial inflammation, apoptosis, and dysfunction. Infection, hypoxia, oxidative stress, environmental toxins, intracellular mediators, and external signals trigger and influence this process [10]. These signals include transforming growth factor beta (TGFβ); interleukin 10 (IL-10); interleukin 1 (IL-1) receptor agonist; and high-density lipoprotein cholesterol (HDL-C), anti-inflammatory cytokines along with vast expression of angiotensin-converting enzyme 2 (ACE2) receptors within endothelial cells, which can cause SARS-CoV-2-binding endothelial compartments; fusion to membrane; viral entry; infection; vascular injury; and disequilibrium and dysfunction of the endothelial environment [10]. Thus, endothelial cell injury and endothelium play a central role in vasculitis associated with COVID-19, which can be evidenced by the presence of inflammatory cells and viral inclusions within the endothelium in histological specimens [10]. This vascular disease can involve multiple organ systems simultaneously, encompassing skin, lungs, kidneys, brain, and other organs. Figure 2 demonstrates the triggers, signals, and mediators of COVID-19-associated vasculitis.

Clinical presentation

Vasculitis associated with COVID-19 presents in different ways, which we will discuss from two different perspectives: vascular endpoints and organ-specific presentation features.

First, COVID-19-related primary vasculitis is the mildest form and can affect children and young adults. It can cause cutaneous vasculitis and Kawasaki-like disease, both of which generally have a good prognosis. In contrast, disseminated intravascular coagulation (DIC) occurs in critically ill COVID-19 pneumonia patients and carries a poor prognosis [11]. Another presentation is systemic arterial and venous thrombosis; the former can increase the risk of stroke and other organ-specific ischemia, whereas the latter often co-presents in many patients with critical illnesses. In both cases, immunothrombotic viral ribonucleic acid is thought to be the key mediator [11]. The last entity in this spectrum is the novel vasculitis mimic caused by the embolization from pulmonary veins to systemic arteries, which requires further research to elaborate its characteristics [11].

Second, the systemic presentation of COVID-19 vasculitis commonly includes a skin rash, asymmetric arthralgia, which can be severe, periarticular swelling, abdominal pain, lymphadenopathy, GN, and stroke among other things. The skin rash can manifest in various ways, such as an erythematous non-pruritic blotchy rash, a diffuse erythematous maculopapular rash, a diffuse rash, and palpable purple papules [10]. Other autoimmune and rheumatologic manifestations of COVID-19, where autoimmunity plays a significant role, will be summarized system-wise in Table 3.

**Table 3:** Organ-specific autoimmune manifestation of COVID-19 This table was prepared as a summary of the mentioned references [6,12]. This table was not directly taken from any reference sources.

Organ System	Manifestation
Brain and nervous system	Encephalitis, myelitis, Guillain-Barré syndrome
Eye	Optic neuritis
Lungs	Interstitial lung disease
Bones and joints	Arthralgia and acute arthritis
Kidneys	Crescentic and collapsing glomerulopathy
Stomach and intestine	Inflammatory bowel disease flare
Blood and lymphatics	Immune thrombocytopenic purpura and autoimmune hemolytic anemia, hemophagocytic lymph histiocytosis
Others	Antiphospholipid antibody syndrome

Differential diagnoses

Granulomatosis with polyangiitis (GPA) was the provisional diagnosis before the biopsy report because the patient exhibited malaise, dyspnea, positive C-ANCA antibodies, nephrotic range proteinuria, and hematuria, all of which improved after solumedrol administration. Prerenal azotemia was another differential diagnosis considered but was ruled out as the BUN:Cr ratio was <20, fractional excretion of Na was >1%, and Cr levels increased despite intravenous hydration. Acute tubular necrosis was also considered but deemed less likely, given no epithelial cells were found in the urine. We considered COVID-19-associated nephropathy due to the nephrotic range proteinuria and AKI, but it was ruled out based on the biopsy results and positive ANCA antibodies.

Diagnosis

Scientists categorize ANCA-associated vasculitis diagnosis into three subclasses based on disease presentation and laboratory results: GPA, microscopic polyangiitis, and eosinophilic granulomatosis with polyangiitis (EGPA). For GPA diagnosis, symptoms should include GN or rapidly progressive GN, pulmonary and gastrointestinal hemorrhage; other organ system involvement; and necrotizing vasculitis of arterioles, capillaries, venules, and perivascular infiltration of inflammatory cells on histology [8]. Laboratory findings should include positive myeloperoxidase-ANCA, positive CRP, proteinuria, hematuria, elevated BUN, and serum Cr [8].

The diagnosis of GPA requires E symptoms (e.g., purulent rhinorrhea, epistaxis, eye pain, visual disturbance, otalgia, and otitis media, pharyngeal ulcer, and hoarseness); L symptoms (e.g., blood in sputum, dyspnea, and cough); K symptoms (e.g., hematuria, proteinuria, rapidly progressive renal failure, hypertension, and edema), and general signs such as fever (temperature of 38-degrees centigrade or higher for two weeks or longer); weight loss; purpura; arthritis; mononeuritis multiplex; ischemic heart disease; necrotizing granulomatous vasculitis with giant cells at the sites of E, L, and K; necrotizing crescentic GN without immune deposits; and necrotizing granulomatous vasculitis of arterioles, capillaries, and venules on histology [8].

Patients with EGPA (Churg-Strauss syndrome) typically present with asthma as a majority symptom. Patients may also exhibit transient and patchy pulmonary infiltrates, allergic rhinitis, sinus polyposis, peripheral neuropathy, essentially mononeuritis multiplex, palpable purpura, urticarial nodule, abdominal pain, bleeding, and kidney involvement showing focal segmental GN with necrotizing features, including crescents [13]. Histologically, Churg-Strauss syndrome shows small vessel angiitis and necrotizing granulomas of extravascular location; clinically, necrotizing vasculitis, eosinophil tissue infiltration, and extravascular granulomas do not often coexist [13].

Treatment

Different treatment options are available for COVID-19-associated vasculitis treatment. Therapy should be based on the symptom, type, and severity. We have summarized other treatment options in Table 4 [14-17].

**Table 4:** Treatment options of COVID-19-associated vasculitis

COVID-19-Associated Vasculitis Treatment	
1.	Most cases self-resolve
2.	Analgesics such as acetaminophen for joint pain
3.	Compression therapy and antihistamine for cutaneous symptoms of immunoglobulin A vasculitis
4.	Steroids should be used if there is a chance of incipient necrosis
5.	High-dose intravenous immunoglobulin and aspirin for Kawasaki disease
6.	Sometimes, it self-resolves. Leg elevation and antihistamines can be used for leukocytoclastic vasculitis
7.	Four to six weeks of tapering corticosteroids for resistant cases
8.	Rarely immunosuppressive therapy and antivirals (e.g., Azathioprine, Hydroxychloroquine, Tocilizumab, Chloroquine, Rituximab, and Favipiravir)
9.	Convalescent plasma or immunoglobulins can be used.
10.	Pneumococcal vaccination
Other measures: social distancing, hand hygiene, droplet precaution, mask, regular follow-up, and completion of influenza	

Complications, prognosis, and risk factors

Acharya et al. suggested using biomarkers and scoring systems for risk stratification of COVID-19 patients. They noted that older age, a high “Sequential Organ Failure Assessment” score, and a D-dimer level of more than 1 mg/mL were associated with a poor prognosis [18]. Among the patients who died, significant risk factors were older age, comorbidities, low oxygen tension, high CRP, low lymphocytes, and complications such as acute cardiac injury, acute respiratory distress syndrome, shock, and DIC [18]. According to the results of a binational, registry-based cohort study, respiratory failure was the most common complication, evident in 54% of patients, followed by AKI in 18%, and secondary infection in 15% of patients [19]. Clinically, 91% of patients needed hospitalization; among them, 11% were admitted to the intensive care unit and 28% of the patients died, whereas the median hospital course length was 11 days, and 38% of patients experienced severe outcomes [19]. Patients with coexisting respiratory illnesses or who were already using corticosteroids had a statistically significant risk of severe consequences [19].

According to Banerjee et al., factors such as female sex, older age, immunosuppression due to any cause, and lung disease were of greater concern for complications [20]. Among the 662 patients studied, 66%, 46%, and 40% avoided doctor visits, laboratory tests, and other tests, respectively; younger age, higher income, and urban location were more prevalent among those who avoided doctor visits and tests [20].

## Conclusions

In this case report, we presented the clinical details of an elderly patient with preexisting comorbidities. The initial treatment focused on addressing respiratory symptoms associated with COVID-19, followed by the subsequent identification and management of ANCA-related vasculitis. Although COVID-19-related complications are common, some may lead to fatality. We hope that this case report will help healthcare professionals make well-informed decisions regarding the likelihood and severity of COVID-19 complications within specific patient populations. Lack of availability of COVID-19-associated vasculitis was a limitation of this study. Also, this was a single case in the hospital; if we could find more cases at the same time, we could compare the cases and present more information. We also propose continued research in this area to refine treatment strategies and improve outcomes for vulnerable populations.
